# Evaluation of optical quality in pterygium patients with a new modified sutureless and glue-free method

**DOI:** 10.1186/s12886-021-02180-2

**Published:** 2021-11-27

**Authors:** Li Peng, Shu Zhou, Bin Yan, Baihua Chen

**Affiliations:** 1 Department of Ophthalmology, The Second Xiangya Hospital, Central South University, Changsha, 410011 Hunan China; 2 Hunan Clinical Research Center of Ophthalmic Disease, Changsha, 410011 Hunan China

**Keywords:** Pterygium, Glue-free, Optical quality, Optical quality analysis system

## Abstract

**Background:**

Modified sutureless and glue-free method is an effective and novel surgical approach for pterygium. We aim to evaluate optical quality in pterygium treated with the new method and investigate the clinical application of the Optical Quality Analysis System (OQAS) and Anterior segment optical coherence tomography (AS-OCT) to evaluate the pterygium surgery.

**Methods:**

A total of 52 eyes of 52 patients with pterygium were randomly divided into 2 groups. After surgical excision, the bare sclera was placed with a tight fit limbal conjunctival autograft fixed via the modified sutureless and glue-free method in group 1 (26 eyes) and conventional sutures in group 2 (26 eyes). Objective scattering index (OSI), modulation transfer function (MTF) and Strehl ratio (SR) were measured using OQAS in both groups during the perioperative period. Pterygium diameter was measured on AS-OCT. Ocular surface disease index (OSDI) questionnaire also was used.

**Results:**

The group 1 had significantly lower mean OSI, higher mean MTF, and higher mean SR at 1 month and 3 months after surgery (*p* < 0.05). The group 1 had significantly lower mean OSDI at 1 month (*p* < 0.05), while was similar to group 2 at 3 months (*p* > 0.05). Pterygium diameter positively correlated with OSI (*r* = 0.528, *p* < 0.001), while it negatively correlated with MTF (*r* = − 0.501, *p* < 0.001) and SR (*r* = − 0.174, *p* = 0.217) before operation.

**Conclusions:**

The modified sutureless and glue-free method might be more advantageous in improving the optical quality during early postoperative recovery times and pterygium diameter affected optical quality. OQAS can be reliably used to evaluate postoperative outcomes.

## Introduction

Pterygium is a benign proliferative lesion that grows from the bulbar conjunctiva to and over the cornea affecting visual function and eventually leads to blindness [1]. Surgical excision of pterygium followed by a conjunctival autograft is widely adopted and has few complications with low recurrence rates [2]. Conjunctival autograft has been conventionally fixed with sutures, which can prolong surgical time and cause ocular discomforts. Thus, there has been an increased enthusiasm among the ophthalmologists to use fibrin glue or autologous blood to simplify the surgical procedure and reduce patient discomfort successfully [3–6]. Recently, we have reported a modified sutureless, glue-free method to fix the conjunctival autograft, which proved to be effective, time-saving, along with a decreased recurrence rate and improved postoperative comfort [7]. Previous studies have shown that pterygium surgery may affect refractive status of the eye [8], but to date, there is no study on the visual quality of the new modified sutureless and glue-free method.

The ocular surface disease index (OSDI) is a questionnaire developed to assess the symptoms related to dry eye and their impact on visual-related functioning [9]. Literature reported a correlation between pterygium formation and shortened BUT. Unstable tear film may contribute to the initiation of pterygium [10]. However, it is a questionnaire survey with subjectivity and unable to provide a comprehensive and objective evaluation of visual quality. Double pass visual optical quality analysis system (OQAS II) works on the dual-channel technique to collect the imaging results by marking and analyzing the point light source reflection to obtain the point spread function (PSF), which can evaluate visual acuity not only objectively, quickly, and accurately but also non-invasively. It provides comprehensive information on diffraction, intraocular scattering, and higher-order aberrations in the ocular optical system, which not only can evaluate visual quality objectively, quickly, and accurately but also non-invasive [11]. The OQAS is the only device that provides objective and high quality retinal-image, which has been used in clinical practice since 2002. It has been shown to be effective in ocular diseases, with OSI being considered to be the most useful parameter [12]. Moreover, the pterygium diameter (defined as the distance from the nasal scleral spur recess to the pterygium tip) can also be assessed by AS-OCT [13].

This study aimed to evaluate the visual quality of patients undergoing our modified sutureless with glue-free method comparing with the traditional suturing method by examining the changes in the OQAS™ II and OSDI among these patients. Meanwhile, the relationship between the pterygium parameter measured by AS-OCT and visual quality was also explored.

## Methods

### Patients

The study met the tenets of the Declaration of Helsinki and was approved by the research ethics committees of the Second Xiangya Hospital, Central South University. All participants gave voluntary informed consent. The inclusion criteria were all adults (18 to 80 years, no gender restriction) diagnosed with primary pterygium > 2 mm, and who consented and received regular follow-up. Exclusion criteria included patients aged older than 80 years, with pseudopterygium, bilateral pterygium, previous ocular surgery, other eye and mental disorders, and clinically significant systemic diseases. A total of 52 patients (52 eyes) diagnosed with primary nasal pterygium were eligible for this study, with all the subjects had a valid indication for pterygium surgery.

### Pterygium surgical procedure

Patients were randomly assigned into 2 groups: the modified sutureless and glue-free group (*n* = 26, named group 1) and the 10/0 nylon sutures group (*n* = 26, named group 2) through a random number table. After surgical excision, the bare sclera of patients in group 1 had a tight fit limbal conjunctival autograft fixed with the modified sutureless and glue-free method, while patients in group 2 underwent the conventional suturing with 10/0 nylon. A detailed surgical procedure was described in our previous study [7]. The approximate surgical procedures were as follows. Firstly, cut the bulbar conjunctiva and fascia parallel to the sides of the pterygium, separate the conjunctiva and its fibrous hyperplasic tissue, cut the conjunctiva at the neck of the pterygium, separate he fibrous tissue, and retrograde torn from the limbus and the cornea. Secondly, scratch bluntly the corneal surface smooth, measure the size of the bare scleral area, harvest a conjunctival autograft with stem cells from the superior temporal quadrant of the same eye and used to cover the defect area where the pterygium tissue had been removed. Finally, flatten and fix the conjunctival autograft with a strabismus hook, using gentle pressure to squeeze out blood and exudate under the autograft (group 1), or by interrupted 10/0 nylon suture (group 2). Adduct or abduct patient eyes to assess whether the autograft remained in place during ocular movements. Both eyes were wrapped with eye pads for 48 h in all patients. Sutures were removed 1 week after the procedure in group 2. Postoperatively, patients received the same medication (tobramycin 0.3% and dexamethasone 0.1%, S.A. ALCON-COUVREUR N.V., Puurs, Belgium) one drop four times a day for 1 week and gradual tapering of doses over 4 weeks and 0.1% sodium hyaluronate solution four times daily for 1 month. All surgeries were performed by the same experienced ophthalmic surgeon (B.C.), who initially did not know to which group the patient was assigned to until the autograft had been harvested.

### Pterygium grading

Pterygium was classified into three grades based on the system by Tan DT et al. [14]: Grade 1 - episcleral vessels under the pterygium were not detected and clearly distinguished; Grade 3 - episcleral vessels could be obscured totally; Grade 2 - between the two states. The effectiveness and security of the modified sutureless and glue-free surgery had been confirmed in our previous study [7].

### Clinical examination and questionnaire

All participants underwent a complete ocular examination including the OSDI symptoms questionnaire and OQAS. To assess symptoms, participants were first required to complete the OSDI questionnaire prior to the other clinical examinations. The questionnaire included 12 items that comprised of three subscales: ocular symptoms (OSDI-symptoms) contained 5 questions (1.Eyes that are sensitive to light? 2. Eyes that feel gritty? 3. Painful or sore eyes? 4. Blurred vision? 5.Poor vision?), vision-related activities of daily living (OSDI-function) contained 4 questions (1.Reading? 2. Driving at night? 3. Working with a computer or bank machine (ATM)? 4. Watching TV?), and environmental triggers contained 3 questions (Windy conditions? 2. Places or areas with low humidity (very dry)? 3. Areas that are air conditioned?) [15]. Each item was rated on a 0 to 4 points scale:0 indicated none of the time; 1, some of the time; 2, half of the time; 3, most of the time; and 4, all of the time. A total score was assessed on a scale of 0 to 100 by calculating the sum of all items in the scale divided by the number of items, with higher scores indicating greater disability. Data on visual quality were taken from the OQAS II (Visiometrics, Spain), and double-pass retinal images of a point source were recorded and analyzed. Object scatter index (OSI), modulation transfer function cutoff frequency (MTF cutoff), and Strehl ratio (SR) were recorded with the OQAS. The OSI is an objective parameter to estimate intraocular scattering. MTF cutoff is used to express visual quality, which can reflect the influence of scattering and aberration on visual image quality. The Strehl ratio is defined as the ratio of the peak height of the PSF divided by the maximum intensity of PSF in the diffraction-limited of the perfect eye. A higher Strehl ratio confers to a better quality of the vision.

### Calculation of pterygium diameter by anterior segment optical coherence tomography (AS-OCT)

**Fig. 1:**
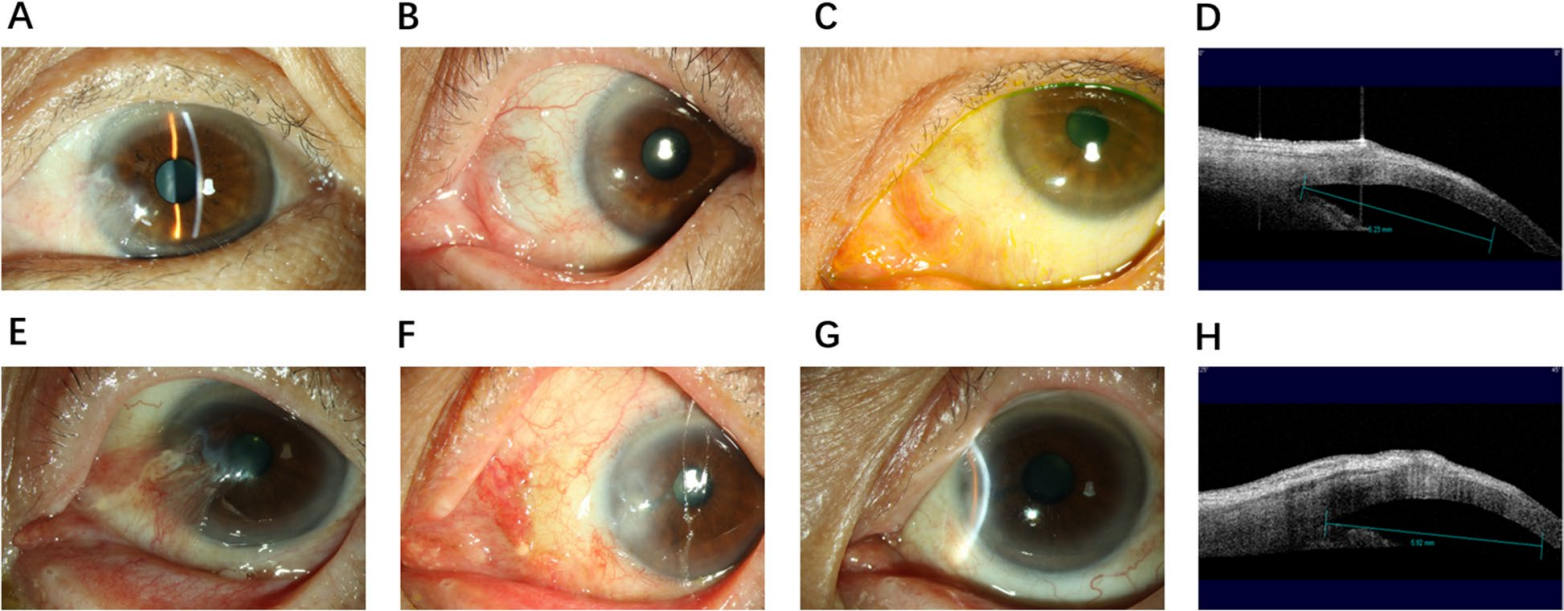
Pre- and postoperative photos and calculation of pterygium size by AS-OCT. Group 1: **A** preoperative, **B** 1 month postoperative, **C** 3 months postoperative, and **D** pterygium diameter, measured as the distance from the nasal angle recess to the pterygium tip (mm). Group 2: **E** preoperative, **F** 1 month postoperative, **G** 3 months postoperative, and **H** pterygium diameter, measured as the distance from the nasal angle recess to the pterygium tip (mm). ↓pterygium tip, the position where high reflection was interrupted

Before surgery, all patients underwent measurement of pterygium diameter by AS-OCT. Pterygium length (horizontal dimension) was defined as the distance from the nasal scleral spur recess to the pterygium tip (mm) (Fig. 1). The pterygium tip was the position where high reflection was interrupted. Each patient was assessed three times by the same operator with the same instrumentation and an average of the three was taken for quantitative analysis.

### Statistical analysis

Demographic data and clinical characteristics were expressed with descriptive statistics (mean ± standard deviation (SD)). After normality testing for continuous variables, outcome variables were compared between groups using the independent t test, or nonparametric Mann–Whitney U test. After Mauchly’s test of sphericity, one-way repeated measure analysis of variance, or followed Greenhouse–Geisser or Huynh–Feldt correction were applied to test the differences at different time-points within groups. One-way ANOVA followed by Tamhane’s T2 test was used to detect differences between OQAS parameters and the pterygium grading. Correlation index (Pearson test) was calculated to evaluate the pterygium diameter and optical quality. A *P*-value of < 0.05 was considered statistically significant. We used the SPSS 22.0 (IBM SPSS: IBM SPSS Statistics Version 22.0) and R statistical analysis package (version 3.5.3) to statistically analyze the data.

## Results

### Clinical characteristics at baseline

Both the modified sutureless glue-free group and the 10/0 nylon suturing group included 26 eyes of 26 patients, respectively. The patient characteristics and optical quality at baseline were illustrated in Table 1. There was no statistically significant difference in the sex, mean age, pterygium diameter, and optical quality between the two groups preoperatively (*p* > 0.05).

**Table 1:** Clinical characteristics and the parameters of optical quality between the two groups at baseline

Characteristics	Group1	Group2	***P***-Value
Eyes (n)	26	26	
Age (years)	58.65 ± 10.32	61.65 ± 7.98	0.246
Male:female	9:17	13:13	
OSI (OSI value)	4.88 ± 3.18	6.22 ± 4.41	0.346
MTF cut-off (c/deg)	16.52 ± 9.23	13.81 ± 10.38	0.229
Strehl ratio	0.12 ± 0.04	0.11 ± 0.03	0.314
pterygium diameter	4.33 ± 1.32	4.50 ± 1.16	0.706
OSDI	21.74 ± 13.67	22.41 ± 10.68	0.491
Pre-pterygium grading (grade 1:2:3)	5:10:11	6:9:11	

### The outcomes of optical quality analysis system (OQAS)

The outcome assessment at each postoperative follow-up was presented in Table 2. Comparative analysis of the two groups was performed to assess the optical quality in the postoperative recovery period at 1 month and 3 months postoperatively. One month after pterygium surgery, patients in both groups showed improvement in optical quality compared with preoperative values. Compared with group 2, group 1 had significantly lower mean OSI (2.16 ± 1.56 vs 3.42 ± 1.87; *p* = 0.005), higher mean MTF cutoff (29.71 ± 12.03 vs 23.16 ± 9.00; *p* = 0.04), and higher mean Strehl ratio (0.19 ± 0.04 vs 0.14 ± 0.04; *p* < 0.001) at 1 month after the operation. Furthermore, Group 1 also had a significantly lower mean OSI (1.64 ± 1.52 vs 2.58 ± 1.43; *p* = 0.004), higher mean MTF (37.94 ± 9.94 vs 33.36 ± 8.31; *p* = 0.039), and higher mean Strehl ratio (0.23 ± 0.05 vs 0.18 ± 0.08; *p* < 0.001) at 3 months postoperatively. These results suggested a higher optical quality in the modified sutureless and glue-free group during the early recovery period. The results of repeated analysis of variance at different time-points within the group showed that the OSI, MTF and SR were differences both within the group1(OSI, *F* (1.095, 28.481) = 35.088, *p* = 0.000. MTF, *F* (2, 52) = 90.128, *p* = 0.000. SR, *F* (1.683, 43.755) = 87.328, *p* = 0.000) and group 2 (OSI, *F* (1.058, 26.451) = 15.833, *p* = 0.000. MTF, *F* (2,50) = 84.704, *p* = 0.000. SR, *F* (1.060,26.489) = 31.259, *p* = 0.000), which suggested a pterygium excision can provide visual quality improvement, regardless of which kind of surgical approach was performed.

**Table 2:** Comparison of optical quality parameters (OSI, MTF cut-off and Strehl ratio) and OSDI scores between and within the two groups after surgery

Optical quality		Presurgery	Postsurgery (1 mo)	Postsurgery (3 mo)	***P***-Value
OSI (OSI value)	Group1	4.88 + 3.18	2.16 ± 1.56	1.64 ± 1.52	*p*1 = 0.00 *p*2 = 0.00 *p*3 = 0.00
	Group2	6.22 + 4.41	3.42 ± 1.87	2.58 ± 1.43	*p*1 = 0.00 *p*2 = 0.00 *p*3 = 0.00
	*P*-value	0.346	0.005	0.004	
MTF cut-off (c/deg)	Group1	16.52 + 9.23	29.71 ± 12.03	37.94 ± 9.94	*p*1 = 0.00 *p*2 = 0.00 *p*3 = 0.00
	Group2	13.81 + 10.38	23.16 ± 9.00	33.36 ± 8.31	*p*1 = 0.00 *p*2 = 0.00 *p*3 = 0.00
	*P*-value	0.227	0.042	0.039	
Strehl ratio	Group1	0.12 + 0.04	0.19 ± 0.04	0.23 ± 0.05	*p*1 = 0.00 *p*2 = 0.00 *p*3 = 0.00
	Group2	0.11 + 0.03	0.14 ± 0.04	0.18 ± 0.08	*p*1 = 0.00 *p*2 = 0.00 *p*3 = 0.00
	*P*-value	0.313	< 0.001	< 0.001	
OSDI	Group1	21.74 + 13.67	10.33 ± 6.23	4.94 ± 3.70	*p*1 = 0.00 *p*2 = 0.00 *p*3 = 0.00
	Group2	22.41 + 10.68	13.88 ± 5.84	6.02 ± 3.14	*p*1 = 0.00 *p*2 = 0.00 *p*3 = 0.00
	*P*-value	0.49	0.037	0.13	

*p*1 Pre vs 1mo, *p*2 1mo vs 3mo, *p*3 pre vs 3mo within group

### The ocular surface disease index (OSDI)

The preoperative mean OSDI scores in group 1 and group 2 were similar (21.74 ± 13.67 and 22.41 ± 10.68, respectively, *p* = 0.49). At 1 month postoperatively, the mean OSDI score was significantly higher in group 2 (13.88 ± 5.84) compared with group 1 (10.33 ± 6.23) (*P* = 0.037). However, the mean OSDI score showed no statistical difference between the two groups at 3 months after surgery (*P* = 0.13).

### Pterygium grading and optical quality

**Fig. 2:**
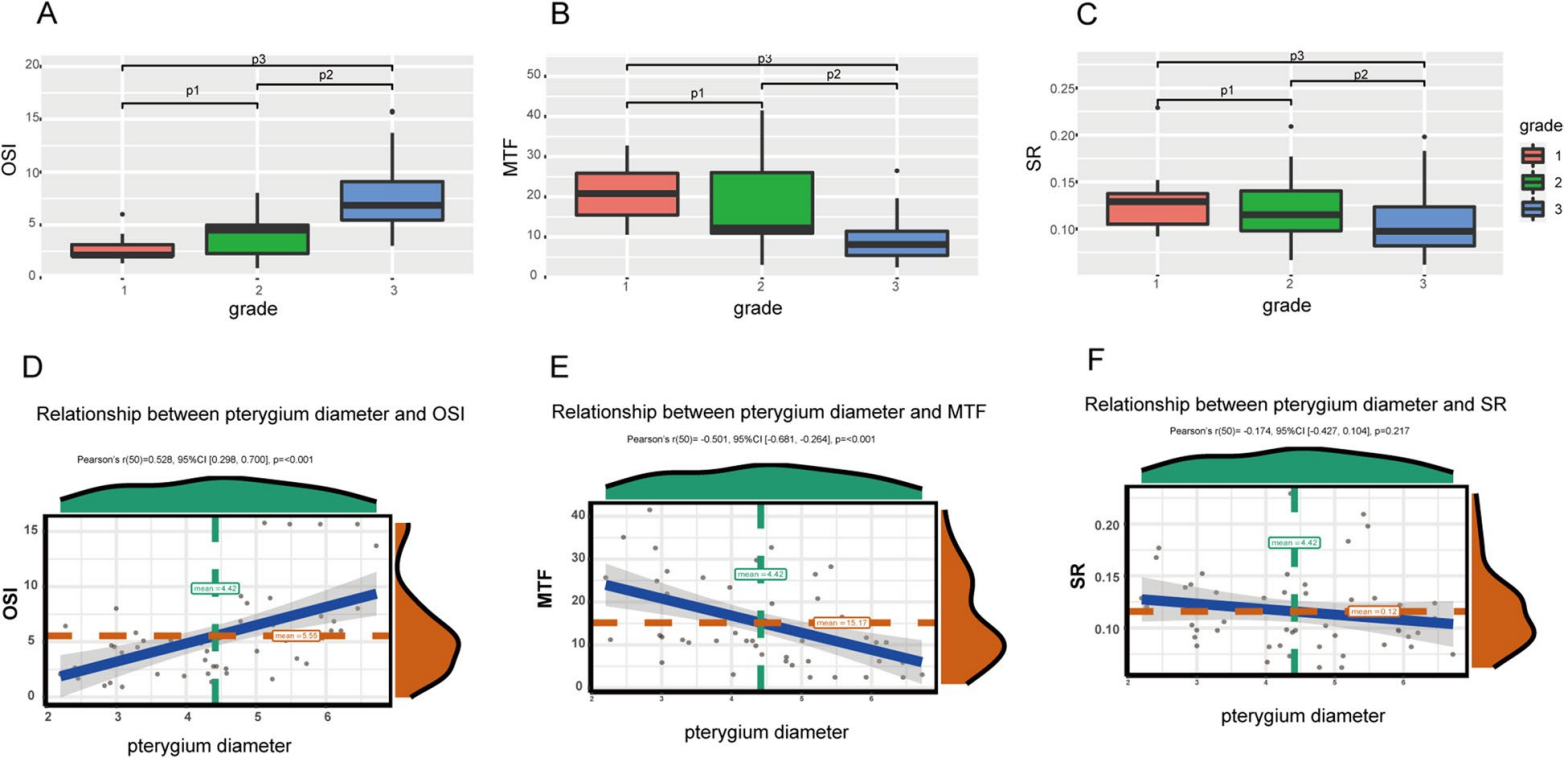
Differential analyses between pterygium grading and three optical quality values (**A**, **B**, **C**), and correlation analyses between pterygium diameter and three optical quality values before surgery (**D**, **E**, **F**). *p*1 Grade 1 vs Grade 2, *p*2 Grade 2 vs Grade 3, *p*3 Grade 1 vs Grade 3

There was no significant difference in the pterygium grading between the two groups before surgery. One-way ANOVA followed by Tamhane’s T2 test was used to detect differences between OQAS parameters and the pterygium grading. The results were illustrated in Fig. 2 and Table 3. Patients in grade 3 showed higher mean OSI (8.37 ± 4.16 vs 2.76 ± 1.34; *p* = 0.01) and lower mean MTF cutoff (9.17 ± 6.15 vs 21.16 ± 7.48; *p* = 0.001) compared with patients in grade 1 before operation. There was no difference in the optical quality parameters between patients in grade 1 and grade 2 (Fig. 2 A, B and C). These findings indicated that higher pterygium grades correlated with worse optical quality.

**Table 3:** Differential analyses between pterygium grading and three optical quality values

	Grade 1 (*n* = 11)	Grade 2 (*n* = 19)	Grade 3 (*n* = 22)	*F*	*p*
OSI (OSI value)	2.76 ± 1.34	3.89 ± 1.94	8.37 ± 4.16	17.048	0.000
	*p1* = 0.201	*p2* = 0.000	*p3* = 0.000		
MTF cut-off (c/deg)	21.16 ± 7.48	18.64 ± 10.93	9.17 ± 6.15	9.893	0.000
	*p1* = 0.843	*p2* = 0.007	*p3* = 0.001		
Strehl ratio	0.13 ± 0.038	0.12 ± 0.037	0.10 ± 0.035	1.996	0.147
	*p1* = 0.515	*p2* = 0.168	*p3* = 0.069		

*p1* Grade 1 vs Grade 2, *p2* Grade 2 vs Grade 3, *p3* Grade 1 vs Grade 3

### Pterygium diameter and optical quality

The overall preoperative mean pterygium diameter was 4.41 ± 1.233 mm. Before surgery, all patients correlated positively with OSI (*r* = 0.528, *p* < 0.001, Pearson’s test), but correlated negatively with MTF (*r* = − 0.501, *p* < 0.001) and SR (*r* = − 0.174, *p* = 0.217) (Fig. 2 D, E and F). The optical quality of pterygium decreased with the diameter of pterygium.

## Discussion

The OSI, MTF cutoff, Strehl ratio (SR) parameters are used for evaluating the optical quality in OQAS. A lower OSI value indicates improved optical quality, a lower value of the MTF cutoff indicates a worse optical quality of the eye, and a higher SR confers to a better quality of the vision. In our study, compared with the suturing group, the modified sutureless and glue-free group demonstrated significantly lower mean OSI, higher mean MTF, and higher mean SR at 1 month and 3 months after surgery, and it had significantly lower mean OSDI at 1 month, while this was similar to the suturing group at 3 months. These results have confirmed that the modified sutureless and glue-free method can improve the visual quality of patients in the early postoperative stage, with the outcomes measured both objectively and subjectively.

The dual-channel visual quality analysis system (OQAS II) developed by Visiometries of Spain provides comprehensive information on diffraction, intraocular scattering, and higher-order aberrations in the ocular optical system [11]. The early clinical application of the OQAS has focused on the area of refractive error and cataract, which is especially valuable for the evaluation of cataract surgery [16]. Several previous studies have acknowledged the importance of visual quality in ophthalmic diseases [17, 18], and optical quality and visual function are affected by aging [18]. Currently, the OQAS is commonly applied when evaluating numerous ophthalmic conditions such as age-related macular disease, diabetic macular edema, retinal vein occlusion [12], dry eye disease [15, 19], central serous chorioretinopathy [20], and primary open-angle glaucoma [21]. However, few studies have reported the use of OQAS in pterygium.

The overall results of our study have shown that all OQAS parameters have been significantly improved at the postoperative follow-ups, indicating the improvement of the visual quality. The pterygium modifies adversely the characteristics of the ocular surface and cornea, resulting in optical quality undermined. Pterygium growth can lead to irregular astigmatism, corneal scarring, restriction of ocular motility, or chronic ocular surface inflammation [22]. Moreover, pterygium has significant influence on high-order aberrations [23]. All of these factors have a great influence on optical quality. A previous study has confirmed that pterygium results in a deterioration in the visual quality [24], and excision can lead to significant improvement that is consistent with our findings. Besides this, our study has demonstrated that the visual quality of the modified sutureless and glue-free group was superior to that of the suturing group both at 1 month and 3 months after surgery. Our previous studies revealed that the average conjunctival autograft thickness were 577 ± 287 mm, 353 ± 159 mm, 185 ± 74 mm in group 1 and 658 ± 205 mm, 408 ± 243 mm, 183 ± 142 mm in group 2 at 1 week, 1 month, 3 months postoperative, respectively. However, no significant differences were found between groups at 1 week or at 1 and 3 months postoperative [7]. The modified surgery method required minimal operating time results in lower trauma and light inflammatory reaction. Improvement of the visual quality may be mainly related to shorter operating time, less inflammation and tear film tear film stability. This suggested that the modified sutureless method has clinical advantages over the more widely used 10/0 nylon sutures method currently, likely because the ocular surface irritation and dry eye symptoms have been significantly alleviated in the modified sutureless group.

A recent study has shown that the OSDI questionnaire has a good correlation and also consistent with patients’ subjective symptoms [25]. Using a standardized OSDI questionnaire, our study has demonstrated that the modified sutureless and glue-free treatment resulted in a statistically significant improvement in patients’ symptoms compared with the suturing method at 1 month after surgery in the early postoperative period, but no difference has been observed at 3 months. Moreover, without the friction of the tied sutures, this method leads to improved postoperative patient comfort. It may enhance the recovery of tear film stability, decrease the tear film scattering, and improve visual quality in pterygium patients. Conventionally, surgical sutures, as a foreign body, can induce more inflammatory reactions which may influence the visual quality in the early postoperative stage. Furthermore, suture removal can cause pain results in greater postoperative discomfort. All of these may explain the lower OSDI score in our study.

The grade of pterygium correlates with the visual quality, with higher grade associated with lower visual quality, as demonstrated in our study. Compared with the modified sutureless and glue-free group, the suturing group had worse visual quality, which may suggest the impact of different surgical treatments on the outcomes of optical quality assessment. The OSDI score, as a questionnaire-based survey, may have some drawbacks because it relies on the patient’s subjective self-reporting. Meanwhile, the OQAS is a valuable tool for an objective evaluation together with all the optical information provided on one surface that is rarely influenced by subjective factors. Our study has suggested that the clinical application of OQAS may be useful in evaluating the visual quality of both preoperative and postoperative patients undergoing treatments for pterygium. Our study contributes new knowledge to the field with a novel method of surgery for pterygium. Nevertheless, there were several limitations to our study. First, the follow-up time was relatively short. Second, the sample size was rather small, and quantitative indices of inflammation and tear film should be more considered during the follow-up. Therefore, a large-scale and stringently-controlled clinical trial with longer follow-up is warranted to validate our findings from this study.

## Conclusions

In conclusion, our modified no suture, glue-free technique is safe and effective for the treatment of primary pterygium requiring surgical resection, with a higher optical quality after surgery compared with the conventional technique. The Optical Quality Analysis System is valuable in assessing the optical quality in patients undergoing treatment for pterygium.

## Data Availability

The data used to support the findings of this study are available from the corresponding author upon request.

## Abbreviations

OQAS: Optical Quality Analysis System
AS-OCT: Anterior segment optical coherence tomography
OSI: Objective scattering index
MTF: Modulation transfer function
SR: Strehl ratio
OSDI: The ocular surface disease index
